# Glucocorticoid regulation of cancer development and progression

**DOI:** 10.3389/fendo.2023.1161768

**Published:** 2023-04-18

**Authors:** Stuti Khadka, Sara R. Druffner, Benjamin C. Duncan, Jonathan T. Busada

**Affiliations:** Department of Microbiology, Immunology and Cell Biology, West Virginia University School of Medicine, Morgantown, WV, United States

**Keywords:** glucocorticoids, inflammation, steroid hormones, cancer development, corticosteroids, endocrine

## Abstract

Glucocorticoids are steroid hormones that regulate a host of cellular and physiological functions. However, they are arguably best known for their potent anti-inflammatory properties. Chronic inflammation is well-known to promote the development and progression of numerous types of cancer, and emerging evidence suggests that glucocorticoid regulation of inflammation affects cancer development. However, the timing, intensity, and duration of glucocorticoid signaling have important but often contradictory effects on cancer development. Moreover, glucocorticoids are widely used in parallel with radiation and chemotherapy to control pain, dyspnea, and swelling, but their use may compromise anti-tumor immunity. This review will explore the effects of glucocorticoids on cancer development and progression with particular focus on pro and anti-tumor immunity.

## Background

In 1938, Compound E was synthesized from the adrenal cortex of beef cattle (1). Within the next decade, adrenal cortical hormones (glucocorticoids or corticosteroids) were used to successfully treat inflammatory diseases such as rheumatoid arthritis and rheumatic fever. Since then, glucocorticoids have risen to be one of the most commonly prescribed drugs in the world. Within the clinic, glucocorticoids are used to treat a host of conditions and diseases, ranging from improving lung development in premature infants, treating allergic reactions, autoimmune diseases, and chronic inflammatory diseases, to anti-cancer therapies. However, despite their widespread clinical use, the mechanisms whereby glucocorticoids regulate diverse cellular and physiological functions have yet to be fully elucidated.

The glucocorticoid receptor (NR3C1 or GR) is expressed by most nucleated cell types in humans. Glucocorticoids are reported to regulate up to 20% of the total transcriptome (2). However, the response to glucocorticoids varies between different cells and tissues. Similar functional diversity of glucocorticoid signaling impacts cancer, and glucocorticoids may inhibit or promote carcinogenesis depending on the cancer type. In addition, the effect of glucocorticoid signaling changes as cancers develop and progress. In some cases, glucocorticoid signaling may suppress damaging inflammation thereby protecting from neoplasia development, but these protective roles are reduced or may reverse during chronic inflammation. Moreover, despite the unclear roles of glucocorticoid signaling in solid tumors, these hormones are commonly co-administered with anti-cancer therapeutic regimens to relieve adverse side effects such as pain, nausea, and dyspepsia and to improve appetite. This review will provide an update on the current knowledge of how glucocorticoid signaling affects cancer development in the pre-tumor microenvironment and how their roles shift in mature tumors. Moreover, we will discuss how glucocorticoids interact with estrogens and androgens in breast and prostate cancer, respectively.

## Steroidogenesis

**Figure 1:**
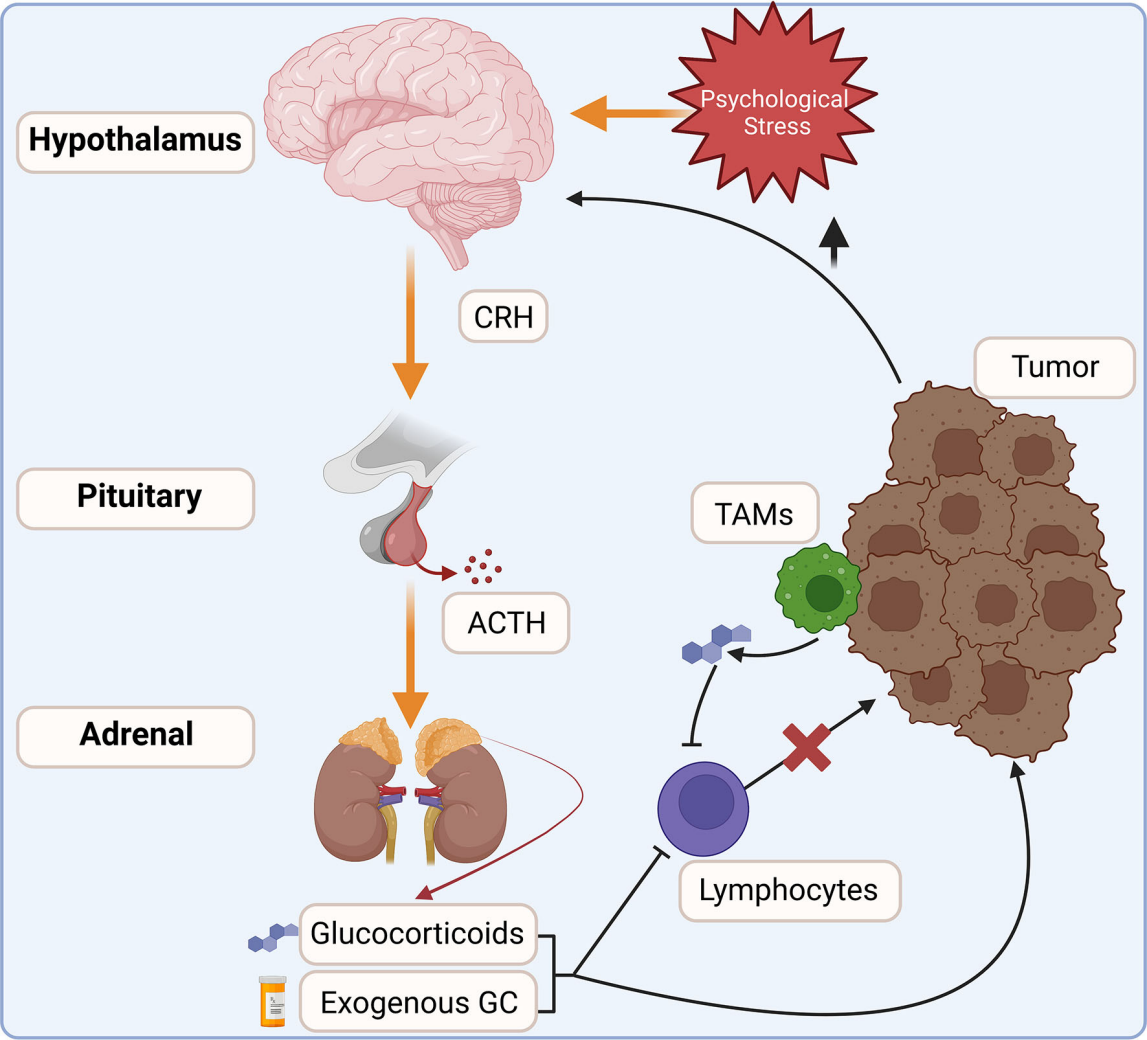
The HPA axis integrates internal and external cues to modulate glucocorticoid production. Increased glucocorticoid signaling is commonly associated with cancers. Increased stress and tissue damage act on the hypothalamus to stimulate the release of corticotropin-releasing hormone (CRH), which in turn stimulates adrenocorticotropic hormone (ACTH) release into circulation. ACTH acts on steroidogenic cells within the adrenal cortex to increase endogenous glucocorticoid production. Exogenous corticosteroids are also commonly co-administered with anti-cancer therapies. Within the tumor, infiltrating leukocytes, such as tumor-associated macrophages (TAMs), activate steroidogenesis and may increase glucocorticoid concentrations within the tumor. Elevated glucocorticoids inhibit anti-tumor immune responses and are linked to increased tumor cell survival.

Glucocorticoids are steroid hormones produced as the end products of the hypothalamic-pituitary-adrenal (HPA) axis. They are produced in a circadian rhythm and in response to psychological and physical stress (**Figure 1**). Glucocorticoid production is initiated in the hypothalamus with the stimulation of neurons within the paraventricular nucleus. This signals the release of corticotropin-releasing hormone (CRH), which acts on the anterior pituitary to promote the synthesis of proopiomelanocortin by corticotropic cells (3, 4). POMC is then cleaved into adrenocorticotropic hormone (ACTH) and released into circulation, acting upon steroidogenic cells within the zona fasciculata of the adrenal cortex. ACTH activates an enzymatic cascade whereby cholesterol is converted into corticosterone (mouse) or cortisol (human) and released into circulation (5, 6). While the HPA axis is well-known to be activated in a diurnal pattern and in response to stress, proinflammatory cytokines and pathogen activated-pattern recognition receptors also trigger glucocorticoid synthesis (7, 8). Upon their release into circulation, glucocorticoids inhibit further activation of the HPA axis. Acute activation of the HPA axis is necessary to suppress immune hyperactivation. However, chronic glucocorticoid signaling is associated with numerous negative health outcomes, such as elevated circulating cytokine levels, chronic inflammation, impaired wound healing, and many other maladies (9). Prolonged glucocorticoid signaling is especially relevant in cancer, where chronic pro-neoplastic infections, cancer-associated psychological stress, corticosteroid therapies, or intra-tumor steroidogenesis inhibit anti-tumor immune responses and promote tumor cell survival (10).

Adrenal steroidogenesis controls systemic glucocorticoid production, but several factors contribute to local glucocorticoid availability. Approximately 80% of endogenous glucocorticoids in the blood are bound by corticosteroid-binding globulin (SERPINA6), which aids transport and regulates bioavailability (11). Proteases such as neutrophil elastase can degrade corticosteroid-binding globulin increasing glucocorticoid concentrations at sites of inflammation (12). Myeloid-derived suppressor cells commonly found within the tumor microenvironment express abundant neutrophil elastase and may utilize this mechanism to increase the glucocorticoid concentration within the tumor. At the tissue level, glucocorticoid concentrations are regulated by hydroxysteroid 11-beta dehydrogenases (HSD11B1 and HSD11B2). HSD11B1 catalyzes the conversion of inactive cortisone into cortisol, thereby increasing local glucocorticoid concentrations. HSD11B1 is widely expressed in the liver, lungs, brain, stomach, adipose tissue, adrenal cortex, uterus, ovaries, and testes (13–15). HSD11B2 catalyzes the reverse reaction by converting cortisol to cortisone and is expressed in the kidneys, colon, and salivary glands (13, 16). These tissues are critical sites of mineralocorticoid receptor signaling, which has a higher binding affinity for glucocorticoids than for its endogenous ligand aldosterone. Thus, HSD11B2 protects the mineralocorticoid receptor from activation by glucocorticoids. Tumor cells are reported to alter the expression of these enzymes, thereby modifying glucocorticoid signaling within the tumor microenvironment (17). For instance, glucocorticoid signaling opposes the development of estrogen-dependent breast cancers, and breast cancers downregulate the expression of HSD11B1 reducing glucocorticoid availability within the tumor (18). Similarly, expression of HSD11B2 is suppressed in skin cancers, increasing intra-tumor glucocorticoid concentrations and promoting invasive and metastatic phenotypes (19).

While the adrenal glands are the primary source of circulating glucocorticoids, increasing evidence suggests that local glucocorticoid production is also important for modulating the functions of healthy tissues and the tumor microenvironment. Extra-adrenal glucocorticoid production primarily exerts paracrine effects (20). Within the intestines, glucocorticoid production by cells within the crypts affects intestinal barrier function and inflammation (21). Within the thymus, nurse cell derived-glucocorticoids raise the threshold for T cell negative selection by suppressing the transcription factors NUR77 and HELIOS (21, 22). Within the tumor microenvironment, glucocorticoids produced by tumor-associated macrophages and tumor-infiltrating T cells promote the expression of T cell checkpoint proteins and inhibit anti-tumor immunity (23–25). Numerous other tissues and cell types are reported to express steroidogenesis enzymes. However, more work is needed to understand the full extent of extra-adrenal glucocorticoid synthesis and its effects on cellular and tissue function.

## Glucocorticoid signaling

**Figure 2:**
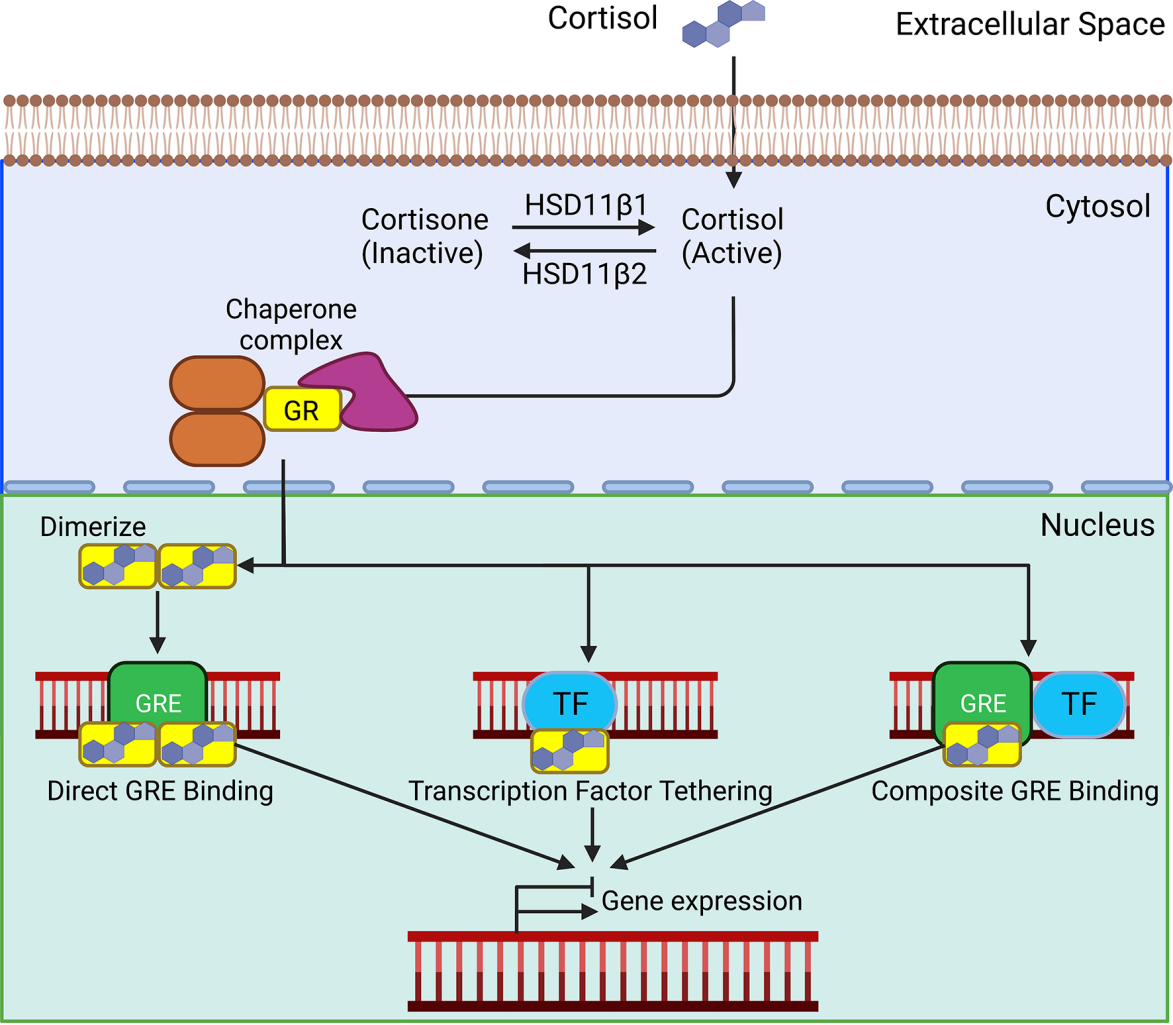
In the absence of ligands, the glucocorticoid receptor (GR) is bound in the cytoplasm by a complex of chaperone proteins. Lipophilic glucocorticoids freely defuse through the plasma membrane and bind to the GR. Upon ligand binding, the GR translocate to the nucleus. Homodimerized GRs bind to glucocorticoid response elements (GREs). Direct binding of the GR to transcription factors (TF) and composite binding to GREs and TF affect their transcriptional activity. These interactions ultimately act to activate or suppress gene transcription.

Glucocorticoids signal through the GR, a member of the ligand-dependent transcription factor superfamily. Structurally, the GR is similar to the other steroid hormone receptors. The GR protein is comprised of three functional domains: an N-terminal transactivation domain, a central DNA binding domain, and a C-terminal ligand binding domain (26). The transactivation domain interacts with coregulators and the transcriptional machinery. The DNA binding domain contains two zinc-finger motifs involved in binding to glucocorticoid response elements (GREs). The DNA binding domain and ligand binding domain are separated by a hinge region that is involved in receptor dimerization and contains two nuclear localization signals, NL1 and NL2. The ligand binding domain contains a hydrophobic pocket for glucocorticoid binding, which induces a conformational change (27). The GR protein also contains several sites for post-translational modifications including, phosphorylation, sumoylation, acetylation, and ubiquitination that regulate receptor activity and turn-over (28). In the absence of ligand, the GR is sequestered in the cytoplasm by a large protein complex comprised of chaperones PTGES3, HSP90, and HSPA1B, and the immunophilins FKBP5 and FKBP4 (**Figure 2**) (27). While bound in this complex, the ligand binding domain of the GR is readily accessible, but the nuclear localization signals are masked. Lipophilic glucocorticoids diffuse freely through the plasma membrane to bind cytosolic GRs. Upon ligand binding, the GR undergoes a conformational change, disassociating the protein complex, and exposing the nuclear localization signals. The cellular effects of this complex dissociation are two-fold; ligand-bound GR is rapidly transported into the nucleus, activating the classical genomic actions of glucocorticoid signaling. Unbound chaperone proteins engage with various other cellular pathways affiliated with some of the non-genomic effects of glucocorticoid signaling (29).

Once in the nucleus, the classical mechanism through which GR affects gene transcription is by binding to GREs, distinct palindromic consensus sequences separated by a 3-5 nucleotide spacer. Binding occurs through the central zinc finger DNA-binding domain, which induces a conformational change within the receptor. Subsequent allosteric interactions promote the recruitment of coactivator complexes necessary to remodel chromatin. These complexes are the cAMP response element binding protein (CREB)/p300 and pCAF, response element-binding protein (CREB)-binding protein/p300 and p/CAF, both of which contain intrinsic histone acetylase activity (30). These proteins acetylate lysine residues to induce nucleosome rearrangement and DNA unwinding, which leads to promoter accessibility and the recruitment of the basal transcription machinery, resulting in target gene transcription. GR monomers can also directly bind to other transcription factors, modifying their activity, or undergo composite interactions requiring both GRE binding and protein interactions with another transcription factor (**Figure 2**) (29). GR can also repress gene activation by binding as a monomer to negative GREs (31). The net effect of these interactions is to induce or suppress gene transcription.

Glucocorticoids are pleiotropic steroid hormones that exert a wide array of effects, such as embryological development, metabolism, cellular proliferation, apoptosis, inflammation, and cancer development (27, 29, 32). Some reports estimate glucocorticoids regulate up to 20% of transcribed genes (2). However, the effects of glucocorticoid signaling are cell-type specific, owing to their interactions with numerous transcription factors, differential chromatin accessibility, and non-genomic effects. The GR mRNA also contains 7 internal translation start sites, and each GR translational isoform has unique transcriptomes (33, 34). Moreover, interactions between the GR and other transcription factors can provide cell-specific effects on transcription. These interactions and cell-type specific effects are important within cancer, where glucocorticoids have significantly divergent roles depending on the cancer type. More work is needed to uncover the mechanisms regulating cell-type specific actions of glucocorticoids both in healthy tissues and in cancer.

## Glucocorticoid regulation of inflammation within the pre-tumor and tumor microenvironments

**Figure 3:**
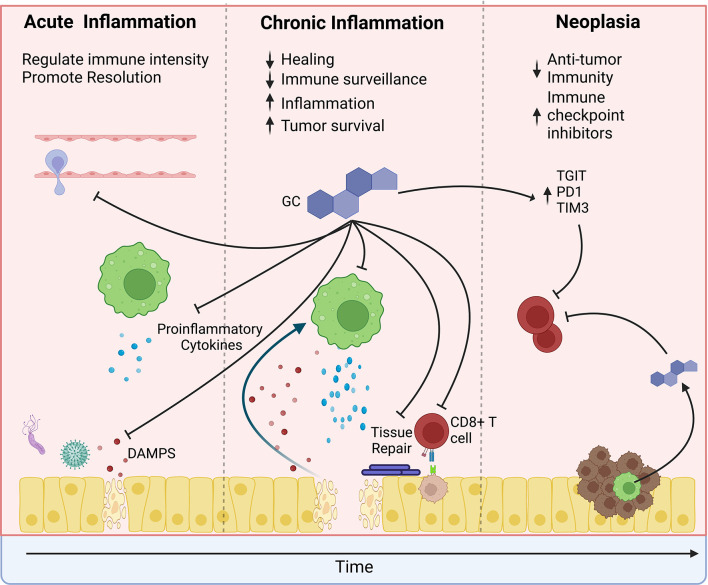
The effects of glucocorticoids change over the course of tumor development. In the pre-tumor microenvironment, glucocorticoids may protect from cancer by limiting the intensity of inflammation. Glucocorticoids suppress immune cell recruitment to sites of damage or infection and suppress proinflammatory cytokine production. However, during chronic inflammation, glucocorticoids may promote tumor development. Prolonged glucocorticoid signaling inhibits wound healing, thereby causing a feedforward loop of increased release of damage-associated molecular patterns (DAMPs), which increase inflammation and further promote tissue damage. Simultaneously, glucocorticoids suppress anti-tumor surveillance by CD8 T cells. Similarly, glucocorticoids suppress anti-tumor immunity within the tumor microenvironment and promote the expression of immune checkpoint proteins. However, their effects are compounded by increased psychological stress, exogenous steroid treatment, and local steroidogenesis by tumor-infiltrating immune cells. Furthermore, glucocorticoids may directly promote tumor cell survival by suppressing apoptosis and promoting tumor cell growth.

The relationship between the immune system and cancer is complex. The immune system is a frontline defense against cancer development. Natural killer cells and CD8+ cytotoxic T cells remove nascent cancer cells with altered MHC I expression or displaying mutated proteins (35, 36). Anti-cancer therapies such as immune checkpoint blockade (ICB) rely on increasing anti-tumor immune activity by stimulating T cells. However, inflammation is a double-edged sword that can also promote cancer development and progression (37). Immune responses to infections and or exposure to irritants, such as inhaled particles, inevitably cause damage to the surrounding tissue. During a “normal” inflammatory response, the pathogens are quickly cleared, the inflammation resolves, and the damaged tissue is healed (**Figure 3**). However, chronic “smoldering” inflammation and the resultant prolonged tissue damage leads to metaplasia development and may promote carcinogenesis. Inflammation associated with chronic infections such as *Helicobacter pylori* (gastric cancer) and Human Papillomavirus (HPV, cervical cancers), chronic inflammatory diseases (chronic obstructive pulmonary disorder (COPD) and Crohn's Disease), and through chronic exposure to irritants such as tobacco use (oral and lung cancers) promote cancer development (38–40). At therapeutic doses, glucocorticoids exert powerful anti-inflammatory effects and have become one of the most prescribed drug classes worldwide. However, severe side effects associated with long-term use limit their effectiveness in treating chronic inflammatory diseases. Glucocorticoids suppress the recruitment of additional leukocytes to the site of inflammation by blocking the production of chemokines, histamine, and prostaglandins and by reducing the expression of adhesion molecules such as integrins and selectins (32). Glucocorticoids also limit the expression of proinflammatory cytokines such as IL1B, TNF, INFG, and IL6 while promoting the expression of anti-inflammatory cytokines such as IL10 and TGFB. Dysregulation of these inflammatory processes is associated with cancer development (41).

Approximately 15-20% of all cancers are preceded by infection (37, 42, 43). Reduced GR expression and polymorphisms that decrease GR activity have long been associated with an increased risk of various cancers. However, the exact stage of cancer development affected by glucocorticoid signaling is unclear. Gastric cancer is the most common pathogen-associated cancer (44). Approximately 90% of gastric cancers are associated with long-term *Helicobacter pylori* infection. Chronic inflammation is a requisite driver of *H. pylori-*associated gastric cancer. Immune-deficient mice are resistant to *Helicobacter-*associated gastric pathologies (45). Moreover, overexpression of the proinflammatory cytokines IL1β or INFγ is sufficient to drive gastric cancer development even in the absence of *Helicobacter* infection (46, 47), underscoring that inflammation is both necessary and sufficient to drive gastric carcinogenesis. Disruption of glucocorticoid signaling is associated with an increased risk of gastric cancer (48). Given their anti-inflammatory effects, glucocorticoids likely suppress pro-neoplastic gastric inflammation. Recently, we reported that adrenalectomized mice developed spontaneous gastric inflammation stemming from the activation of type 2 innate lymphoid cells (ILC2s) and the recruitment of monocyte-derived macrophages (49, 50). Adrenalectomy-induced gastric inflammation drove atrophic gastritis and pyloric metaplasia development, which are precursors of gastric adenocarcinoma. It is important to note that the adrenalectomy-induced gastric inflammation and pre-neoplastic lesions occurred without *Helicobacter* infection, suggesting that glucocorticoids are master regulators of gastric inflammation. While these studies show an essential role for glucocorticoids in protecting the stomach from pro-neoplastic inflammation, excessive glucocorticoid signaling may compromise protective gastric immunity. For instance, treatment with the synthetic glucocorticoid dexamethasone during *H. pylori* infection protects from pre-neoplastic epithelial changes but also increases gastric bacterial load (51). Excessive glucocorticoid signaling is also linked to peptic ulcer development (52). Thus, the magnitude and duration of glucocorticoid signaling likely contribute to how these hormones affect carcinogenesis. Further investigation is needed to uncover how glucocorticoid signaling during *H. pylori* infection affects gastric cancer development.

The lung is also highly susceptible to inflammation-associated neoplasia. Lung inflammation caused by tobacco smoking, particle inhalation, or exposure to gases such as ozone is linked to lung cancer development (53). While tobacco smoking is the top risk factor for lung cancers, smokers with chronic lung inflammatory diseases such as COPD are 3-10 fold more likely to develop lung cancer than smokers without COPD (54). In mouse mutant KRAS lung cancer models, lung inflammation accelerates tumor formation. However, depleting discrete inflammatory cell populations or reducing the concentration of proinflammatory cytokines protects from tumor development (55–57). Similarly, in urethane lung tumor models, glucocorticoid treatment suppressed lung tumor development while adrenalectomy significantly increased tumor number (58, 59). Glucocorticoid treatment also reduced cancer risk in mice exposed to cigarette smoke (60). Therefore, glucocorticoid treatments that reduce lung inflammation protect from cancer development. Within the clinic, glucocorticoids are a frontline therapy for treating lung inflammatory diseases (61). Epidemiological studies have found that inhaled steroid use dramatically reduces the risk of lung cancer in individuals with lung inflammation, such as COPD (62, 63) and asthma (64). Thus, glucocorticoids may be critical in suppressing pro-neoplastic lung inflammation.

Glucocorticoid signaling within the pre-tumor microenvironment is a double-edged sword. While glucocorticoid signaling suppresses pro-neoplastic inflammation and limits tissue damage, they may also activate processes that promote neoplasia development (**Figure 3**). Glucocorticoids inhibit wound healing by suppressing collagen deposition, growth factor expression, and vascularization (65). These delays in wound healing may increase inflammation, epithelial proliferation, and metaplasia, promoting carcinogenesis. Tang et al. reported that while deletion of the GR from the intestinal epithelium increased acute inflammation, intestinal GR knockout mice exhibited accelerated tissue healing and fewer intestinal tumors than GR wild type mice (66). Similarly, treatment with the GR agonist betamethasone suppressed acuteintestinal inflammation but also prevented epithelial healing promoting tumor development. Therefore, sustained glucocorticoid suppression of tissue repair promotes chronic inflammation and may promote cancer development. In addition to preventing wound healing, glucocorticoid signaling also suppresses anti-cancer immune surveillance by CD8+ cytotoxic T cells (67). Thus, as mutations occur, glucocorticoids may suppress the removal of developing cancer cells.

Cancer cell escape from immune surveillance is critical for tumor formation, survival, and growth. Glucocorticoids potently suppress anti-tumor immunity. Within the myeloid compartment, glucocorticoids alter the phenotype of tumor-associated macrophages, suppressing their proinflammatory functions while promoting anti-inflammatory roles and driving the production of the anti-inflammatory cytokines TGFB and IL10 (68). Glucocorticoids also curtail the anti-tumor effects of dendritic cells by inducing the expression of *TSC23D3* (*GILZ*), thereby reducing proinflammatory cytokine expression and suppressing antigen presentation to CD8+ T cells (69). Within the lymphoid compartment, glucocorticoids directly suppress CD8+ T cell activation by driving the expression of the immune checkpoint inhibitors TGIT, PD1, TIM3, and LAG3 and through promoting the expression of the anti-inflammatory cytokine IL10 (23). CD8+ T cells are key mediators of anti-tumor immunity, and suppression of these cells through the induction of checkpoint proteins significantly promotes tumor growth and metastasis. Immune-activating therapies such as ICB are designed to suppress these checkpoint proteins and promote anti-tumor immunity. However, elevated expression of GR-regulated genes is associated with reduced ICB responsiveness (23), and high systemic glucocorticoid levels are linked to ICB failure (70, 71). Consistent with their ability to antagonize ICB efficacy, glucocorticoids are the standard treatment for immune-related adverse events associated with ICB (72).

## Glucocorticoid effects on cancer cells and tumor progression

In contrast to their heterogeneous effects on tumor development, glucocorticoid signaling within established solid tumors is typically linked to poor clinical outcomes. Elevated glucocorticoid signaling during cancer occurs through the activation of the HPA axis and treatment with exogenous steroids. In preclinical models, chronic psychological stress suppresses anti-tumor immunity and increases tumor growth (73). Similar outcomes are reported in clinical studies where depression and poor patient mood increase systemic glucocorticoid production and are linked to suppression of anti-tumor immunity and increased mortality (69, 74). Glucocorticoids are also commonly co-administered with chemotherapy to reduce negative side effects such as nausea and edema and to improve appetite and energy (75). Similar to the negative effects of elevated endogenous glucocorticoids, corticosteroid treatment is associated with reduced anti-tumor immune responses, tumor treatment resistance, and increased metastasis. In addition to a systemic increase of adrenal-derived glucocorticoids, tumor-infiltrating immune cells also may produce glucocorticoids within the tumor (10, 23–25). Local steroidogenesis may be an important paracrine mechanism by which tumor cells can evade the immune system, but the overall impact of extra-adrenal glucocorticoid production on tumor progression and patient outcomes remains unknown.

Glucocorticoid signaling in cancer cells is heterogenous, and their impacts depend on the cancer type and stage. Within tumor cells, glucocorticoids promote proliferation, survival, and migration. These pro-tumor effects are manifested through a variety of mechanisms. In some cases, direct GR transcriptional targets are critical for promoting tumor cell survival. For instance, treatment with dexamethasone protects thyroid cancer cells from apoptosis by induction of BCL-XL (76). However, consistent with its high-level role as a transcriptional regulator, many cellular effects are manifested through the induction of gene expression or signaling cascades that ultimately promote tumor progression. This is illustrated by the GR-target glucocorticoid-regulated kinase 1 (SGK1), which promotes tumor cell survival through interacting with the PI3K pathway and through the activation of the Forkhead box family of transcription factors such as FKHRL1 (77). Furthermore, SGK1 phosphorylation of N-MYC downstream regulated gene 1 (NDRG1) promotes tumor cell growth and tissue extravasation. NDRG1 expression is elevated in esophageal squamous cell carcinoma, wherein expression is correlated with local tumor invasion and poor prognosis (78). Additionally, NDRG1 dysregulation in esophageal cancer has pro-oncogenic functions by activating the WNT signaling pathway (79). Similarly, betamethasone promotes increased expression of cytoskeletal proteins such as fibronectin, promoting the activation and nuclear accumulation of the oncoprotein YAP and the expansion of chemotherapy-resistant cancer stem cells (80). Therefore, the effects of glucocorticoids on cancer cells may often be masked by downstream effectors, and there are likely numerous effects of glucocorticoid signaling that have not yet been described.

In addition to their classical transcriptional effects, the GR impacts cancer through rapid non-genomic actions. Within the A549 lung carcinoma cell line, the GR regulates the activation of both WT and mutated RAS through non-genomic actions (81). In the absence of glucocorticoids, cytoplasmic GR directly binds to RAS, inhibiting its activation. GR suppression of RAS blocks tumor cell proliferation, and GR-deficient A549 cell xenografts developed into larger tumors than WT cells which express endogenous GR (81). Importantly, GR suppression of RAS is blocked by treatment with dexamethasone. Thus, increased glucocorticoid levels within the tumor microenvironment would block GR binding to RAS and promote tumor growth.

While glucocorticoid signaling in solid tumors is often linked to poor clinical outcomes, this is not universally true. Patient hepatocellular carcinomas actively suppress glucocorticoid signaling by upregulating HSD11B2. Bypassing the HSD11B1/B2 axis through long-term administration of dexamethasone protects from liver carcinogenesis in mice by suppressing glycolysis and promoting gluconeogenesis (82). Similarly, short-term administration of hydrocortisone attenuates the progression of hepatocellular carcinoma in rats (83). The GR transcriptional target *TSC22D3* suppresses RAS and RAF signaling by directly binding these proteins and preventing the activation of the MAPK pathway and RB phosphorylation (84). Similarly, GR promotes the expression of the phosphatase DUSP1, which inhibits multiple components of the MAPK pathway including, p38 kinase, JNK, and ERK (85, 86). These seemingly conflicting results highlight the cell-type-specific effects of glucocorticoid signaling and the need for further research on the impact of glucocorticoids on cancer.

## Glucocorticoid interactions with other steroid hormone receptors

All steroid hormone receptors primarily function as ligand-dependent transcription factors. Many of the mechanisms utilized to regulate transcription are shared between the steroid hormone receptors. Estrogen and androgen signaling are central players in breast and prostate cancer development and progression, respectively. Glucocorticoids can modulate the activity of these steroid hormones through multiple mechanisms, including heterodimerizing with the estrogen receptor (ER) and androgen receptor (AR) and competing for co-factors (87–89). Moreover, the DNA response elements recognized by each steroid hormone receptor are highly similar, often only differing by a few nucleotides (90). Therefore, certain ER and AR-regulated genes can also be targeted by the GR.

In ER-positive breast cancer, GR expression is correlated to a favorable prognosis (91, 92). Glucocorticoids may inhibit the development of ER-positive tumors by antagonizing the activity of ER alpha. GR is reported to antagonize ER signaling through several distinct mechanisms, such as displacing ER alpha binding to estrogen response elements and by heterodimerizing with the ER to recruit co-repressors (87, 93). GR-ER binding is also associated with increased expression of differentiation markers, inhibition of ER-induced growth factors, and increased survival of patients with ER-positive breast cancer (92). Thus, glucocorticoid signaling may be protective in estrogen-dependent breast cancer. Further supporting this idea, a recent epidemiological study found that systemic glucocorticoid therapies were associated with reduced incidence of ER-positive but not ER-negative invasive breast cancers (94). However, protection was only associated with grade I and II tumors, while glucocorticoid use was associated with an increased risk of metastasis for higher-grade tumors, potentially due to reduced estrogen dependence in more advanced tumors. In contrast to their putative protective roles in early-stage ER-positive breast cancers, glucocorticoids are linked to poor outcomes in patients with ER-negative tumors (91, 92, 95). In these tumors, glucocorticoids are reported to assume a role similar to the ER by inducing the expression of ER-regulated genes, promoting growth and metastasis (80, 96). Further information about the role of glucocorticoids in breast cancer can be found in this cited review article (97).

In prostate cancer, glucocorticoids play important roles in promoting resistance to anti-androgen therapies. Various reports disagree on whether the GR is expressed by healthy human prostate epithelial cells. However, GR expression increases as the tumors acquire resistance to anti-androgen therapies (98, 99). Treatment with AR antagonists is also associated with increased circulating cortisol levels and decreased expression of the cortisol-degrading enzyme HSD11B2 by prostate cancer cells, increasing local cortisol concentrations (100, 101). Exogenous corticosteroids are also commonly co-administered with anti-androgen therapies to alleviate side effects (102). Collectively, these changes promote increased glucocorticoid signaling within prostate cancer cells.

The GR and AR are structurally similar and share nearly identical DNA response elements (90). Cells that co-express the AR and GR have significant overlap in their regulated transcriptomes (98, 103). During anti-androgen therapies, when only the GR is transcriptionally active, the GR drives the expression of typically AR-regulated genes related to apoptotic resistance and tumor cell survival (104, 105). It is important to note that while there is significant overlap between AR and GR-regulated transcriptomes, they are not identical, and each steroid hormone receptor regulates a host of specific genes and cellular functions. However, the similarity between these receptors allows for some functional overlap. Indeed, numerous studies have reported the immunomodulatory roles of androgens (106, 107). We recently reported that glucocorticoids and androgens regulate the transcription of proinflammatory cytokines within ILC2s, suppressing gastric inflammation and metaplasia (50). Therefore, many of the effects of glucocorticoids on anti-cancer immunity could also be influenced by androgens. Interactions between glucocorticoids and androgens likely play critical roles in the development of numerous types of cancers and warrant further investigation.

## Clinical implications

Glucocorticoids are used extensively as cancer therapy adjuvants for the treatment of solid tumors. Synthetic glucocorticoids such as dexamethasone, prednisolone, and betamethasone have long been utilized for improving adverse symptoms associated with chemotherapy and radiation therapy (108–110). Dexamethasone ameliorates chemotherapy-induced cytotoxicity especially in breast and prostate cancer (104, 111). In brain tumors, dexamethasone is used to reduce intracranial pressure and edema (112). Despite their significant use for managing symptoms in solid tumors, corticosteroid use is linked to the failure of anti-tumor therapies and poor patient outcomes. Glucocorticoid treatments are linked to suppression of anti-tumor immunity, ICB failure, cancer cell resistance to chemotherapy, and increased tumor metastases (113–115). In a retrospective study of 181 glioblastoma patients receiving anti-PDL1 therapy, dexamethasone treatment was the strongest predictor for reduced patient survival (71, 116). Corticosteroid treatment as an adjuvant to radiotherapy for glioblastoma has consistently shown a reduction in overall patient survival and promotes tumor recurrence (117, 118). Similarly, in non-small cell lung cancer and urothelial carcinoma, glucocorticoid treatment is associated with reduced patient survival (119).

Despite these host of negative effects, glucocorticoid treatments offer considerable positive benefits. As a chemotherapy adjuvant, glucocorticoids suppress nausea, improve appetite and energy, and reduce pain (75). Suppression of these negative side effects improves patient quality of life and increases patient adherence to oral chemotherapy regimens (120). Corticosteroids are often utilized during tumor resections to reduce anesthesia-related nausea and vomiting and suppress inflammation within the surgical site. Glucocorticoid treatments after resection of Barret’s esophagus or esophageal carcinoma protect from the development of esophageal strictures (121). Retrospective studies have found that intra- and post-operative treatments with corticosteroids have no impact on patient survival or cancer recurrence, demonstrating that carefully constructed therapeutic regimens may be able to deliver the benefits while avoiding the negative complications of glucocorticoid therapies (122, 123). However, the exact effects of glucocorticoids likely vary depending on tumor type, and much more work is needed to fully elucidate how glucocorticoid adjuvant therapies affect various cancer types.

## Conclusions

Glucocorticoid signaling is highly divergent across different cancer types, and their effects significantly change as cancers develop. Despite their widespread clinical use to combat the side effects of chemotherapy, there is a significant gap in our knowledge concerning how glucocorticoid signaling affects specific cancer types. However, even less is known about how glucocorticoids affect early tumor development. Our summary of the literature suggests that glucocorticoids may be a double-edged sword in the pre-tumor microenvironment where they protect from pro-neoplastic inflammation early, but sustained activation may promote carcinogenesis by promoting chronic inflammation and inhibiting wound repair. Significantly more work is needed to improve our knowledge of how these hormones affect cancer development and how the clinical use of glucocorticoids affects anti-cancer treatment efficacy. Finally, interactions between glucocorticoids and other steroid hormones clearly affect breast and prostate cancer, but these interactions likely affect numerous other cancer types. Glucocorticoid and androgen signaling, in particular, shape the anti-tumor T cell response. More work is needed to uncover how interactions between stress and sex hormones affect anti-tumor immunity.

## Author contributions

JB conceived the topic and constructed the original outline. SK, SD, BD, and JB wrote the original manuscript. BD and JB constructed the figures. All authors contributed to the article and approved the submitted version.
